# Retraction: Identification of an immunologic signature of lung adenocarcinomas based on genome-wide immune expression profiles

**DOI:** 10.3389/fmolb.2024.1469693

**Published:** 2024-07-31

**Authors:** Frontiers Editorial Office

The journal retracts the 11 January 2021 article cited above.

Following publication, concerns were raised regarding the integrity of the images in the published figures. Images presented in this article were subsequently also identified in publications from unrelated author groups. Following consultation with the authors during the investigation, which was conducted in accordance with Frontiers’ policies, we have also been unable to confirm authorship by all named authors. As a result, the data and conclusions of the article have been deemed unreliable and the article has been retracted.

This retraction was approved by the Chief Executive Editor of Frontiers. The authors received a communication regarding the retraction and agree to retract the article. This communication has been recorded by the publisher.

Frontiers would like to thank Dr. Sholto David for contacting the journal regarding the published article, the concerns regarding which were also documented on PubPeer.

